# Outcome of Carotid Endarterectomy after Regional Anesthesia versus General Anesthesia - A Retrospective Study Using Two Independent Databases

**Published:** 2014-09-04

**Authors:** Jiabin Liu, Hecter Martinez-Wilson, Mark D. Neuman, Nabil Elkassabany, Edward Andrew Ochroch

**Affiliations:** Department of Anesthesiology and Critical Care, the Perelman School of Medicine, University of Pennsylvania, Philadelphia, PA, USA 19104

## Abstract

**Background:**

Carotid endarterectomy (CEA) is effective in reducing stroke risk in selected patient groups. The ideal anesthetic technique remains controversial in light of literature between general anesthesia (GA) and regional anesthesia (RA) for CEA.

**Methods:**

We studied the NSQIP data from 2005 to 2012. There were 32,718 patients receiving general anesthesia (GA) and 5,384 patients receiving regional anesthesia, local anesthesia, or monitored anesthesia care (RA). The outcome measurements of 30 days postoperative complications were death, stroke, coma, unplanned intubation, on ventilator > 48 hours, cardiac arrest, and myocardial infarction. We next studied NY-SID data from 2007 to 2011. There were 13,913 patients receiving GA and 3,145 patients receiving RA. The outcome measurements by discharge time were death, stroke, paraplegia, new neurological disorder, aspiration, respiratory failure, pulmonary resuscitation procedure (include intubation), cardiac arrest, cardiac resuscitation procedure, myocardial infarction, and congestive heart failure. All analyses were risk adjusted with propensity score matching algorithm.

**Results:**

There were significant differences in incidences of un-expected intubation (1.21% vs. 0.55%, P=0.001), and myocardial infarction (0.80% vs. 0.35%, P=0.039) between GA and RA respectively in NSQIP data. GA group had significant higher incidences of aspiration (0.61% vs. 0.19%, P=0.014), and pulmonary resuscitation procedure (including intubation) (1.02% vs. 0.54%, P=0.044) than RA group in NY-SID data.

**Conclusions:**

In comparison to GA, patients receiving RA had significant lower risks of postoperative unplanned intubation and/or pulmonary resuscitation procedure after carotid endarterectomy.

## Introduction

Carotid endarterectomy (CEA) is effective in reducing stroke risk in selected patient groups. CEA is commonly performed under general anesthesia (GA), regional anesthesia, local anesthesia, or monitored anesthesia care. Based on the typical intraoperative care paradigms, we chose to define regional anesthesia (RA) to include any of the above local anesthetic based anesthesia practice, including regional anesthesia, local anesthesia, and monitored anesthesia care. The choice of anesthesia is largely based on patient factors, surgeon’s preference, and the culture of the institution. The ideal anesthetic technique remains controversial as multiple small studies produced conflicting results regarding the association of GA versus RA with mortality [1-7], stroke [2-8], hemodynamic homeostasis [1,3-5], and cardiac morbidity [3,6,7].

The GALA (general anesthesia versus local anesthesia for carotid surgery) study was the only large randomized controlled clinical trial with 3526 patients, and it concluded that there was no difference in incidences of death, stroke, or myocardial infarction between GA and RA (9). While GALA study provided the most convincing comparisons between GA and RA, it has its limitation. The GALA study reported that 65% of the patients were ASA I or ASA II [9], while other study revealed ~90% of patients undergoing CEA were ASA III or IV [10].

The American College of Surgeons National Surgical Quality Improvement Program (NSQIP) is a nationally validated outcome-based program to measure surgical outcomes. It contains 140 variables, including patient demographic information, preoperative comorbidities, intra-operative variables, and 30-day postoperative complications. A retrospective study by Schechter and colleagues on the NSQIP data from 2005 to 2009 looking at the composite risks of stroke, myocardial infarction, and death did not show significant patient outcome differences between GA and RA groups (2.8% versus 3.6%) undergoing CEA (11). However, Schechter et al. reported significant differences in secondary complications between GA and RA (4.1% versus 2.9%) without detail information on the nature of these differences [11]. Leichtle et al. studied the same NSQIP data from 2005 to 2009 with a propensity matching strategy, and concluded that GA was associated with higher incidence of myocardial infarction (odds ratio 5.41), while no differences were reported for mortality and stroke risks [10].

With the release of NSQIP data from 2010-2012 we proposed to take advantage of the much larger dataset to study low incidence clinically relevant postoperative complications during CEA. We hypothesized that there are no differences on 30-day postoperative central nervous, pulmonary, and cardiovascular system complications between GA and RA patients.

New York State Inpatient Database is another independent database publically available via the US Agency for Healthcare Research and Quality’s (AHRQ) Health Care Utilization Project (HCUP). The database contains information on patient demographic information, International Classification of Diseases-9-Clinical Modification (ICD-9-CM) code for diagnoses, ICD-9-CM code for procedures, anesthesia type, and discharge status. There was no previous study on outcome differences between GA and RA among CEA patients in the NY-SID data. Hereby we propose to utilize the NY-SID data as a replication set to further test our hypothesis.

## Materials and Methods

### Data source

This study was exempted by the institutional review board (the University of Pennsylvania, Philadelphia, Pennsylvania, USA 19104).

NSQIP Data:We acquired the American College of Surgeons National Surgical Quality Improvement Program (NSQIP) database from 2005 to 2012 (http://site.acsnsqip.org). NSQIP members prospectively submit data on 140 variables, which are validated via strict standardized protocol. The data include demographic information, pre-existing comorbidities, intraoperative variables, and postoperative complications for 30 days after the surgery. The full list of information collected is available at NSQIP (http://site.acsnsqip.org/participant-use-data-file/). The NSQIP participant user data files included 152,490 subjects in 2005 and 2006, 211,407 subjects in 2007, 271,368 subjects in 2008, 336,190 subjects in 2009, 363,431 subjects in 2010, 442,149 subjects in 2011, and 543,885 subjects in 2012.

NY-SID Data: We acquired the US Agency for Healthcare Research and Quality’s (AHRQ) Healthcare Cost and Utilization Project (HCUP) New York State Inpatient Database (NY-SID) from 2007 to 2011 (http://www.hcup-us.ahrq.gov). NY-SID includes the collection of all encounter-level information in the state of New York. The data include demographic information, anesthesia type, ICD-9-CM diagnosis code, ICD-9-CM procedure code, AHRQ comorbidity measures of various organs/systems, and discharge status. The full list of information collected is available online (http://www.hcup-us.ahrq.gov/db/state/siddist/sid_multivar.jsp, last accessed August 15, 2014). The NY-SID data included 2,608,615 subjects in 2007, 2,629,383 subjects in 2008, 2,661,905 subjects in 2009, 2,612,610 subjects in 2010, and 2,578,680 subjects in 2011.

### Study sample definition

**Figure 1:**
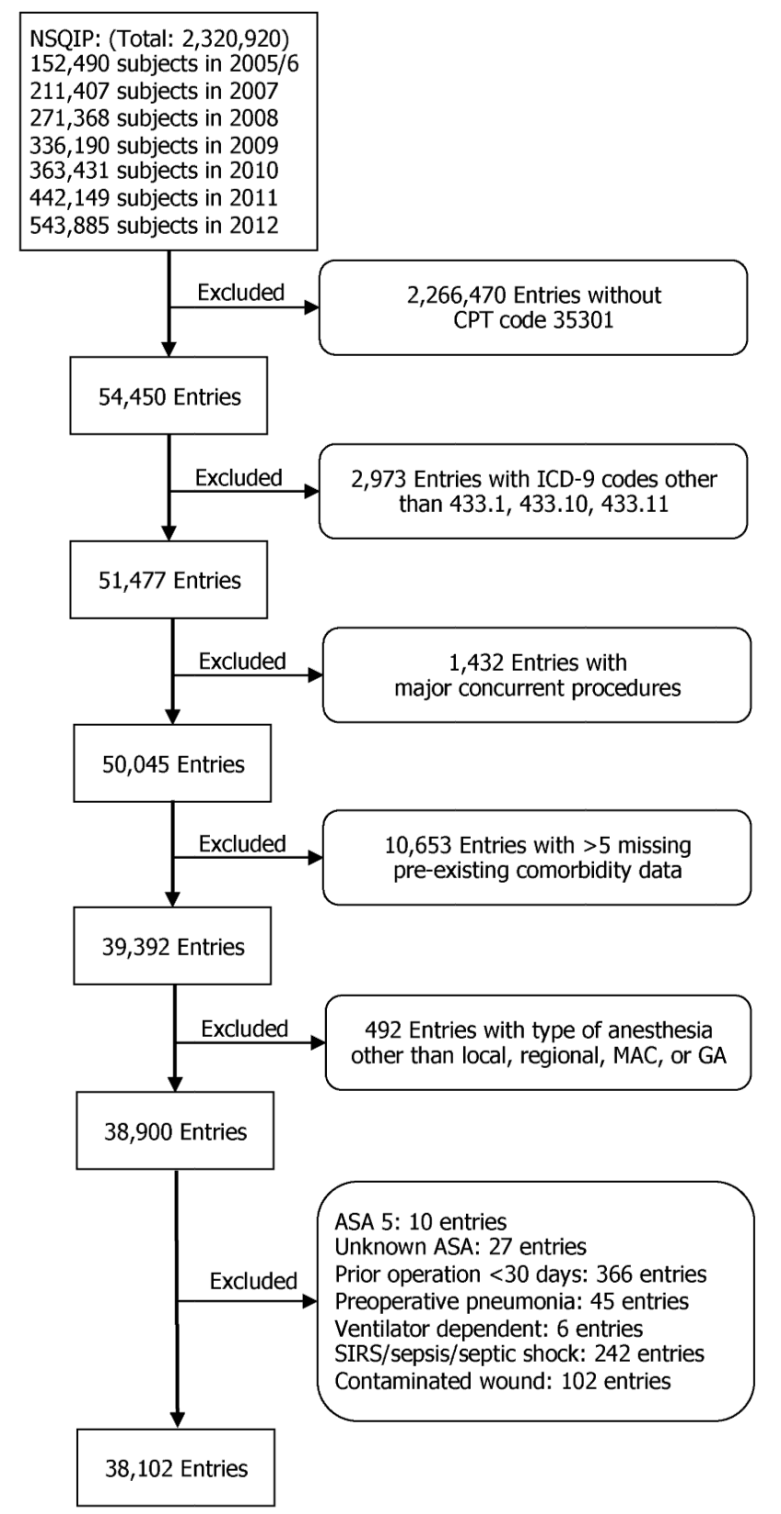
Creation of study sample with NSQIP database. CPT: Current Procedural Terminology. ICD-9: International Classification of Diseases-9. MAC: Monitored Anesthesia Care. GA: General Anesthesia.

NSQIP Study Sample: To define our study cohort in NSQIP data, we included patients with the Current Procedural Terminology (CPT) code for carotid endarterectomy as their principal procedure (CPT code 35301). There were total of 54,450 entries with the listed CPT code as principal procedure. We first removed patients with ICD-9-CM diagnosis other than carotid occlusion and stenosis (ICD-9 433.1, 433.10, or 433.11). We next excluded patients with other significant concurrent procedures as defined by a relevant concurrent CPT code, which could have significantly effects on the choice of anesthesia and postoperative complication rates. (e.g. combined CEA and CABG) We elected to apply work relative value unit >2.11 as the cutoff criteria to eliminate patients with significant concurrent procedures, while maintaining patients with relevant benign procedures that were relevant to carotid endarterectomy (such as angiography, ultrasonography, arterial cannulation, etc.). We then removed entries with more than five missing comorbidity data points. Next, we excluded patients who received anesthesia type other than general, local, regional, or monitored anesthesia care. We also excluded patients with ASA classification 5 (moribund). Last, we excluded patients who had prior operations within 30 days, pneumonia, ventilator dependence, systemic inflammatory response syndrome (SIRS), sepsis, septic shock, or contaminated/infected/dirty wound classification preoperatively. A diagram illustrating the defining process is summarized in figure 1.

**Figure 2:**
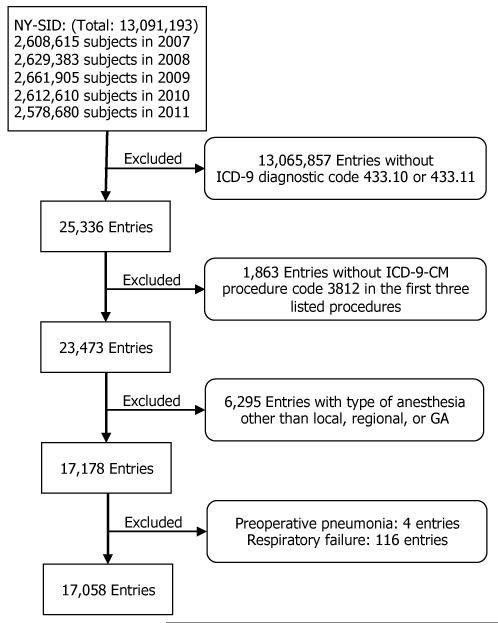
Creation of study sample with NY-SID database. ICD-9: International Classification of Diseases-9. ICD-9-CM: International Classification of Diseases-9 Clinical Modification. GA: General Anesthesia.

NY-SID Study Sample: To define our study cohort in NY-SID data, we included patients with the primary diagnosis of carotid occlusion and stenosis with or without cerebral infarction (ICD-9-CM 433.10 or 433.11). There were total of 25,336 entries. We first removed patients without ICD-9-CM procedure code (3812) in the first three listed procedures. Next, we excluded patients who received anesthesia type other than general, local, or regional anesthesia. Last, we excluded patients who had pneumonia or were ventilator dependent preoperatively. A diagram illustrating the defining process is summarized in figure 2.

### Exposure variable

The NSQIP Participant User Data File coded anesthesia types into the following categories: general, local, regional, monitored anesthesia care, spinal, epidural, other, none, or unknown. In cases where general anesthesia was used concurrently with other type(s) of anesthesia, patients were coded as receiving general anesthesia. For the present study, we grouped patients receiving local, regional, or monitored anesthesia care together in a single category as regional anesthesia.

The NY-SID codes method of anesthesia types into the following categories: local, general, regional, other, none, or unknown. In cases where general anesthesia was used concurrently with other type(s) of anesthesia, patients were coded as receiving general anesthesia. For the present study, we grouped patients receiving local and regional anesthesia together in a single category as regional anesthesia.

### Study variables

The NSQIP dataset contains demographic information (age, gender, height, weight, race), type of anesthesia, American Society of Anesthesiologists (ASA) Physical Status Classification, level of functional dependence prior to surgery in activities of daily living, wound classification, and comorbidities. For this study, we created variables corresponding to the individual system or organ: severe chronic obstructive pulmonary disease (COPD), congestive heart failure, coronary artery disease (defined as history of myocardial infarction, prior percutaneous coronary intervention, previous cardiac surgery, or history of angina in one month before surgery), peripheral vascular disease (defined as history of revascularization/amputation for peripheral vascular disease, or rest pain/gangrene), hypertension requiring medications, diabetes mellitus with or without insulin treatment, end stage liver disease (defined as presence of ascites, or esophageal varices), kidney failure (defined as acute renal failure, or currently on dialysis), central nervous system (CNS) disease (defined as impaired sensorium, coma>24 hours, history of transient ischemic attack, cerebrovascular accident/stroke with or without neurological deficit, or tumor involving CNS), spinal cord injury (defined as hemiplegia, paraplegia, or quadriplegia), and active malignancy (defined as disseminated cancer, chemotherapy, or radiotherapy for malignancy).

The NY-SID dataset contains demographic information (age, gender, race), type of anesthesia, ICD-9-CM diagnosis code, diagnosis present on admission indicator, ICD-9-CM procedure code, and AHRQ HCUP comorbidity measures. The comorbidity measures include congestive heart failure, pulmonary circulation disorders, peripheral vascular disease, chronic pulmonary disease, diabetes, liver disease, renal failure, central nervous system disease, malignancy, and etc. The full list is available at http://www.hcup-us.ahrq.gov/db/state/sasddist/sasd_multivar.jsp; last accessed on Aug 15, 2014. The definition of each comorbidity by ICD-9-CM code is available at http://www.hcup-us.ahrq.gov/toolssoftware/comorbidity/Table2-FY12-V3_7.pdf, last accessed on Aug 15, 2014.

### Outcome variables

We obtained data on six postoperative complications within NSQIP database. These included stroke/CVA, coma > 24 hours, unplanned intubation, on ventilator > 48 hours, cardiac arrest requiring CPR, and myocardial infarction. All six variables were defined as either diagnosed by surgeon or attending physician, or on the basis of pre-defined clinical and laboratory criteria as specified at NSQIP website.

We were able to obtain data on 10 postoperative complications within NY-SID database. These included stroke, paralysis, new neurological disorder, aspiration, respiratory failure, pulmonary resuscitation procedure (including reintubation and extended ventilator support), cardiac arrest, cardiac resuscitation procedure, myocardial infarction, and congestive heart failure. Each variable is identified via ICD-9-CM code algorithms as previously described (12-15), with additional ICD-9-CM coding algorithms on paralysis and new neurological disorder at HCUP website (http://www.hcup-us.ahrq.gov/toolssoftware/comorbidity/Table2-FY12-V3_7.pdf, last accessed on Aug 15, 2014).

### Statistical analysis

A propensity score was calculated with logistic regression modeling that included all study variables. Variables for the NSQIP matching algorithm include age, gender, BMI, race, ASA Status, level of functional status, and all pre-existing comorbidities. Variables for the NY-SID pairing include age, gender, race, and AHRQ comorbidity measures. The propensity score represented the probability of receiving RA for each patient in the range of 0 to 1. The propensity scores were then applied to create two matched groups of GA and RA with the caliper distance of 0.005 without replicates. The matched cohorts were then compared similarly as described above. All data analyses were executed in STATA 12.1 (StataCorp LP, College Station, TX, USA). Fisher’s exact test and chi-square test were used for categorical data. Student T-test and Wilcoxon test were applied for interval data. Statistic significance was defined as P<0.05. Our main focus was to compare individual central nervous, pulmonary, and cardiac system outcomes between GA and RA groups. To compensate the existing differences on patient characteristics and comorbidities between GA and RA groups, we conducted propensity score matching algorithm (described below) to generate two matching groups of GA and RA patients for further analysis.

## Results

### The cohort from the NSQIP database

We identified 54,450 patients who underwent carotid endarterectomy via CPT code between 2005 and 2012 in the NSQIP database (Figure 1). We excluded 2973 patients with concurrent ICD-9 codes other than 433.1, 433.10, or 433.11 (Carotid artery occlusion and stenosis). We then removed 1432 entries with major concurrent procedures, and 10,653 entries with more than 5 missing pre-existing comorbidity data fields. We next excluded 492 patients with types of anesthesia other than local, regional, monitored anesthesia care, or general anesthesia. We further eliminated patients with ASA classification 5 - moribund (n=10), unknown ASA classification (n=27), prior operations within 30 days (n=366), preoperative pneumonia (n=45), ventilator dependent (n=6), systemic inflammatory response syndrome (SIRS)/sepsis/septic shock (n=242), and contaminated/infected/dirty wound classification (n=102). The final cohort contained 38,102 patients (Figure 1).

There were 32,718 (85.87%) GA, and 5384 (14.13%) RA subjects in the NSQIP dataset (Table 1). There were statistical, but probably not clinically, significant differences in age, gender, BMI, and race. The average hospitalization days were 2.47 vs. 2.09 days between GA and RA group (P<0.0001).

The GA group had higher prevalence of ASA class III and IV patients (91.14% vs 90.04%), COPD (10.51% vs 9.51%), diabetes on insulin (9.69% vs 8.64%), and central nervous system disease (43.33% vs 41.18%) than RA group (Table 2). The RA group had more patients with hypertension requiring medication (86.53% vs 85.20%), and spinal cord injury (0.54% vs 0.35%) than GA group. All subjects were then fitted with the propensity score matching analysis, and a subgroup of matched patients (n=4880 per group) from the total cohort was generated for further comparison. Analysis of preoperative demographic information and pre-existing comorbidities indicated covariate balance between GA and RA group (Table 3 and Table 4).

Table 5 lists the incidence of central nervous, pulmonary, and cardiovascular system complications before and after propensity score matching within 30 days postoperatively in the NSQIP data. The overall 30 day mortality was 0.76% and 0.72% between GA and RA subjects respectively (P=0.906). The RA group had lower incidences of unplanned intubation after surgery (0.55% vs 1.21%, P=0.001), and myocardial infarction (0.45% vs 0.80%, P=0.039).

#### The cohort from the NY-SID data

We then set out to replicate these findings in the NSQIP data in the NY-SID data where we identified 25,336 patients with CEA listed within the first three procedures (Figure 2). We then removed 1863 patients without carotid occlusion and stenosis with or without cerebral infarction enlisted as primary diagnosis. Next, we excluded 6295 patients who received anesthesia type other than general, local, or regional anesthesia. Last, we excluded patients with preoperative pneumonia (n=4) and respiratory failure (n=116). The final cohort contained 17,058 subjects (Figure 2).

There were 13,913 subjects in the GA group and 3,145 subjects in the RA group in the NY-SID patient’s database (Table 6). The average length of hospitalization was 2.60 vs 2.05 days between GA and RA group (P<0.001). Further analysis of the comorbidities indicated several differences (Table 7). The GA group had higher prevalence of patients with anemia (5.82% vs 4.29%), rheumatoid arthritis/collagen vascular disease (2.25% vs 1.53%), fluid/electrolyte disorder (5.11% vs 2.77%), and diabetes (32.16% vs 29.86%) than RA group. All subjects were then fitted with the propensity score matching analysis with NY-SID variables, and a subgroup of matched patients (n=3134 per group) from the total cohort was generated for further comparison. Analysis of preoperative demographic information and pre-existing comorbidities indicated the equality between GA and RA group (Table 8 and Table 9).

The overall inpatient mortalities were 0.26% and 0.10% in the GA and RA groups (P=0.226), which were lower than the 30 days mortality rate in NSQIP data as expected (Table 10). RA group was associated with lower incidences of aspiration (0.19% vs 0.61%, P=0.014), pulmonary resuscitation procedure including reintubation and ventilator support after surgery (0.54% vs 1.02%, P=0.044). There was no difference of myocardial infarction (0.61% vs 0.83%, P=0.370) in the NY-SID data.

## Discussion

Our study using prospectively collected NSQIP data of 38,102 CEA patients and NY-SID data of 17,058 CEA patients suggests that regional anesthesia was associated with better outcome indicated by some of the complication indexes.

### Why the current study is needed?

There were many studies comparing general anesthesia and regional anesthesia in CEA. However, most studies were limited by sample size to be conclusive. Meta-analysis could potentially draw conclusions to this debate. However, the heterogeneity of these studies could not adequately power the meta-analysis to draw convincing conclusion. A meta-analysis with 48 studies in 2007, including 14 prospective and 34 retrospective studies (16) concluded that, lower incidences of death, stroke, and myocardial infarction in patients receiving RA despite of the limited study power due to the low number of prospective studies (16). However, the multicenter randomized prospective control trial, GALA trial with total of 3526 patients, found no differences in mortality, stroke, myocardial infarction, or length of hospital stay (9). A recent meta-analysis of 14 trials and 4596 operations noticed there were lower incidences of stroke and mortality in RA group compared to GA group, while the differences were not statistically significant (17). We took advantage of the two available large databases to address this debate on anesthesia type and surgical outcome in CEA.

Our results support these previous reports that there were no differences in mortality and stroke risks between GA and RA groups. NSQIP data showed significant lower incidence of myocardial infarction. However, NY-SID data did not support the hypothesis of lower incidence of myocardial infarction. This is likely due to the fact that NY-SID database only included ICD-9-CM code information to the point of hospital discharge, and thus potentially missed later onset complications, such as myocardial infarction, which were routinely monitored in the NSQIP data collection process. Nonetheless, our analysis on two large databases with 38,102 and 17,058 CEA operations provided valuable information on incidence of myocardial infarction at a much larger scale.

Pulmonary complications have not been well studied. Our analysis indicated differences in risks of unplanned intubation after surgery between GA and RA in the NSQIP data. Our analysis also verified the lower incidence of unplanned intubation/prolonged ventilator support in the RA group in the NY-SID data. Furthermore, NY-SID data indicated RA group is associated with lower incidence of aspiration risk. Unfortunately, the incidence of aspiration could not be studied in the NSQIP data due to lack of such information. To our best knowledge, this is the first study with special focus on pulmonary complications. While the overall mortality and stroke risks were similar between GA and RA patients, these secondary postoperative complications could have significant implications on the requirement of perioperative resources, the length of hospitalization, and the quality of life of patients. The difference might cast significant socioeconomic influence on the patient and health care system.

### Limitations of the study

The authors acknowledge that the conclusion of this study is limited due to the retrospective nature of this study design. The coding system of anesthesia type in the NSQIP and NY-SID databases is also a significant limitation. In case of concurrent use of general anesthesia with any other type(s) of anesthesia, patients were coded as receiving general anesthesia. Therefore, patients who were initially planned for RA and converted to GA might represent intraoperative complications that would show up as postoperative complications and thus be miss-assigned with anesthesia type. The NSQIP database also removed hospital and surgeon identification information in order to comply with participation agreement between NSQIP and participating sites. However, this information might be of interest to adjust relative risks. Similarly, there are limitations in the NY-SID database. NY-SID contains encounter-level information, and the integrity of the data relies on the accuracy of ICD-9-CM coding. Although many studies have validated the reliability of ICD-9-CM coding algorithm in outcome studies, the retrospective nature limited the study power.

## Conclusion

Our study showed that regional anesthesia was associated with lower incidences of unexpected intubation and pulmonary resuscitation procedure after CEA compared to general anesthesia. The study of two large independent databases, NSQIP database and NY-SID database, provided more evidence on the potential beneficial effect of regional anesthesia on pulmonary complications among CEA patients. However, the choice of type of anesthesia for CEA should also be based on surgeon’s recommendation and patient’s preference considering the limited benefit with regional anesthesia.

## Figures and Tables

**Table 1:** NSQIP Patient Demographic Information Summary by Anesthesia Type.

		General Anesthesia			Regional Anesthesia			P-value
		N/Mean		%/SD	N/Mean		%/SD	
Total Subjects (N)		32718		85.87%	5384		14.13%	
Age:		70.94	±	9.51	72.15	±	9.32	<0.0001
Gender:	Male	19271		59.00%	3243		60.45%	0.046
	Female	13393		41.00%	2122		39.55%	
	Unknown	54		19				
BMI:		28.13	±	6.63	27.81	±	6.16	0.0009
Race:	White	27572		90.60%	4502		92.24%	<0.001
	Black	1344		4.42%	147		3.01%	
	Hispanic	988		3.25%	151		3.09%	
	Others	529		1.74%	81		1.66%	
	Unknown	2285			503			
LOS		2.47	±	5.31	2.09	±	6.06	<0.0001

All values were reported as mean ± SD, or percentage.

The unit of measure for BMI is kilogram/meter^2^.

**Table 2:** NSQIP Prevalence of Pre-existing Comorbidities.

		General Anesthesia		Regional Anesthesia		
Comorbidity		N	%	N	%	P-value
ASA classification	4-Life Threat	4201	12.84	568	10.55	<0.001
	3-Severe Disturbance	25618	78.30	4280	79.49	
	2-Mild Disturbance	2850	8.71	533	9.90	
	1-No Disturbance	49	0.15	3	0.06	
Functional health status Prior to Surgery	Dependent	97	0.30	10	0.19	0.388
	Partially Dependent	1375	4.21	225	4.18	
	Independent	31221	95.50	5149	95.64	
CHF in 30 days before surgery		258	0.79	41	0.76	0.934
Coronary Artery Disease		11785	36.02	1964	36.48	0.520
Peripheral Vascular Disease		3257	10.01	509	9.45	0.210
HTN requiring medication		27877	85.20	4659	86.53	0.010
Dyspnea	At Rest	346	1.06	52	0.97	0.836
	Moderate Exertion	5596	17.10	927	17.22	
History of severe COPD		3439	10.51	512	9.51	0.026
End Stage Liver Disease		26	0.08	5	0.09	0.795
Renal Failure		376	1.15	49	0.91	0.141
Diabetes Mellitus	Insulin	3172	9.69	465	8.64	0.047
	Oral/Non-insulin	6062	18.53	1005	18.67	
Central Nervous System Disease		14178	43.33	2217	41.18	0.003
Spinal Cord Injury		115	0.35	29	0.54	0.042
Disseminated cancer, Chemotherapy/Radiotherapy		148	0.45	27	0.50	0.587
Bleeding disorders		6723	20.55	1083	20.12	0.477

ASA: American Society of Anesthesiologists; CHF: Congestive heart failure; HTN: Hypertension; COPD: Chronic Obstructive Pulmonary Disease.

**Table 3:** NSQIP Patient Demographic Information Summary by Anesthesia Type in the Propensity Score Matched Sub-groups (N=4880 per group).

		General Anesthesia			Regional Anesthesia			P-value
		N/Mean		%/SD	N/Mean		%/SD	
Age:		72.10	±	9.21	72.16	±	9.36	0.7462
Gender:	Male	3024		61.97%	2956		60.57%	0.164
	Female	1856		38.03%	1924		39.43%	
BMI:		27.86	±	6.12	27.84	±	6.16	0.8542
Race:	White	4502		92.25%	4501		92.23%	0.253
	Black	144		2.95%	147		3.01%	
	Hispanic	131		2.68%	151		3.09%	
	Others	103		2.11%	81		1.66%	

All values were reported as mean ± SD, or percentage. The unit of measure for BMI is kilogram/meter^2^. “Other race” included patients listed as Native Hawaiian or Pacific Islander, Asian or Pacific Islander, Asian, and American Indian or Alaska

Native in the NSQIP database.

**Table 4:** NSQIP Prevalence of Pre-existing Comorbidities in the Propensity Score Matched Sub-groups (N=4880 per group).

		General Anesthesia		Regional Anesthesia		
Comorbidity		N	%	N	%	P-value
ASA classification	4-Life Threat	520	10.66	528	10.82	0.381
	3-Severe Disturbance	3927	80.47	3877	79.45	
	2-Mild Disturbance	432	8.85	472	9.67	
	1-No Disturbance	1	0.02	3	0.06	
Functional health status Prior to Surgery	Dependent	7	0.14	7	0.14	0.975
	Partially Dependent	201	4.12	205	4.20	
	Independent	4672	95.74	4668	95.66	
CHF in 30 days before surgery		34	0.70	38	0.78	0.723
Coronary Artery Disease		1766	36.19	1793	36.74	0.585
Peripheral Vascular Disease		449	9.20	458	9.39	0.780
HTN requiring medication		4241	86.91	4228	86.64	0.720
Dyspnea	At Rest	52	1.09	50	1.02	0.951
	Moderate Exertion	843	17.27	839	17.19	
History of severe COPD		466	9.55	467	9.57	1
End Stage Liver Disease		9	0.18	5	0.10	0.424
Renal Failure		50	1.02	41	0.84	0.400
Diabetes Mellitus	Insulin	421	8.63	430	8.81	0.873
	Oral/Non-insulin	898	18.40	912	18.69	
Central Nervous System Disease		1967	40.31	2015	41.29	0.333
Spinal Cord Injury		25	0.51	24	0.49	1
Disseminated cancer, Chemotherapy/Radiotherapy		30	0.61	26	0.53	0.688
Bleeding disorders		982	20.12	1000	20.49	0.669

ASA: American Society of Anesthesiologists; CHF: Congestive heart failure; HTN: Hypertension; COPD: Chronic Obstructive Pulmonary Disease.

**Table 5:** NSQIP Incidences of 30-days Post-operative Complications before and after Propensity Score Matching.

	Before Propensity Score Matching					After Propensity Score Matching				
	GA(N=32718)		RA(N=5384)			GA(N=4880)		RA(N=4880)		
Variable Label	N	%	N	%	P-value	N	%	N	%	P-value
Mortality	238	0.73	37	0.69	0.795	37	0.76	35	0.72	0.906
Stroke/CVA	481	1.47	77	1.43	0.854	76	1.56	74	1.52	0.934
Coma >24 hours	20	0.06	5	0.09	0.387	1	0.02	5	0.10	0.219
Unplanned Intubation	365	1.12	27	0.50	<0.001	59	1.21	27	0.55	0.001
On Ventilator > 48 Hours	212	0.65	24	0.45	0.091	38	0.78	23	0.47	0.071
Cardiac Arrest Requiring CPR	84	0.26	10	0.19	0.377	9	0.18	10	0.20	1
Myocardial Infarction	257	0.79	25	0.46	0.010	39	0.80	22	0.45	0.039

GA: General anesthesia; RA: Regional anesthesia; CVA: Cerebrovascular accident; CPR: Cardiopulmonary resuscitation.

**Table 6:** NY-SID Patient Demographic Information Summary by Anesthesia Type.

		General Anesthesia			Regional Anesthesia			P-value
		N/Mean		%/SD	N/Mean		%/SD	
Total Subjects (N)		13913		81.56%	3145		18.44%	
Age:		71.49	±	9.43	71.71	±	9.52	0.2433
Gender:	Male	8009		57.56%	1889		60.06%	0.010
	Female	5904		42.44%	1256		39.94%	
Race:	White	11942		86.52%	2797		89.22%	<0.001
	Black	467		3.38%	58		1.85%	
	Hispanic	755		5.47%	107		3.41%	
	Others	638		4.62%	173		5.52%	
LOS		2.60	±	4.91	2.05	±	3.14	<0.001

All values were reported as mean ± SD, or percentage. “Other race” included patients listed as Asian or Pacific Islander, Native American, or other in the NY-SID database.LOS: Length of Hospital Stay in days.

**Table 7:** NY-SID Patient Demographic Information Summary by Anesthesia Type in the Propensity Score Matched Sub-groups (N=3134 per group).

		General Anesthesia			Local Anesthesia			P-value
		N/Mean		%/SD	N/Mean		%/SD	
Age:		71.62	±	9.39	71.70	±	9.53	0.7224
Gender:	Male	1883		60.08%	1883		60.08%	1
	Female	1251		39.92%	151		39.92%	
Race:	White	2779		88.67%	2796		89.22%	0.916
	Black	63		2.01%	58		1.85%	
	Hispanic	112		3.7%	107		3.41%	
	Others	180		5.4%	173		5.52%	

All values were reported as mean ± SD, or percentage. “Other race” included patients listed as Asian or Pacific Islander, Native American, and other in the NY-SID database.

**Table 8:** NY-SID Prevalence of Pre-existing Comorbidities in the Propensity Score Matched Sub-groups (N=3134 per group).

	General Anesthesia		Regional Anesthesia		
Comorbidity	N	%	N	%	P-value
Alcohol	49	1.56	48	1.53	1
Deficiency Anemia	117	3.73	135	4.31	0.274
Rheumatoid Arthritis / Collagen Vascular Disease	48	1.53	46	1.47	0.917
Chronic Blood Loss Anemia	9	0.29	10	0.32	1
Congestive Heart Failure	140	4.47	162	5.17	0.215
Chronic Pulmonary Disease	642	20.49	657	20.96	0.663
Coagulopathy	21	0.67	25	0.80	0.658
Drug Abuse	3	0.10	6	0.19	0.507
Hypertension	2563	81.78	2564	81.81	1
Liver Disease	25	0.80	29	0.93	0.682
Fluid / Electrolyte Disorders	73	2.33	87	2.78	0.298
Obesity	185	5.90	196	6.25	0.597
Paralysis	0	0	4	0.13	0.125
Peripheral Vascular Disorders	560	17.87	574	18.32	0.670
Pulmonary Circulation Disorders	32	1.02	34	1.08	0.902
Renal Failure	219	6.99	238	7.59	0.382
Valvular Disease	231	7.37	246	7.85	0.505
Weight Loss	4	0.13	8	0.26	0.387
Mental Disorder	199	6.35	213	6.80	0.508
Cancer	43	1.37	49	1.56	0.600
Diabetes	922	29.42	938	29.93	0.678

Mental disorder: includes depression and psychoses; Cancer: includes lymphoma, metastatic Cancer, and solid tumor without metastasis; Diabetes includes insulin dependent and insulin independent diabetes;

**Table 9:** NY-SID Prevalence of Pre-existing Comorbidities.

	General Anesthesia		Regional Anesthesia		
Comorbidity	N	%	N	%	P-value
Alcohol	163	1.17	48	1.53	0.108
Deficiency Anemia	810	5.82	135	4.29	0.001
Rheumatoid Arthritis/ Collagen Vascular Disease	313	2.25	48	1.53	0.011
Chronic Blood Loss Anemia	34	0.24	11	0.35	0.333
Congestive Heart Failure	749	5.38	163	518	0.693
Chronic Pulmonary Disease	2987	21.47	660	20.99	0.563
Coagulopathy	139	1.00	25	0.79	0.313
Drug Abuse	49	0.35	6	0.19	0.167
Hypertension	11444	82.25	2573	81.81	0.553
Liver Disease	89	0.64	29	0.92	0.095
Fluid / Electrolyte Disorders	711	5.11	87	2.77	<0.001
Obesity	771	5.54	196	6.23	0.135
Paralysis	27	0.19	4	0.13	0.642
Peripheral Vascular Disorders	2405	17.29	575	18.28	0.185
Pulmonary Circulation Disorders	137	0.98	34	1.08	0.620
Renal Failure	1022	7.35	239	7.60	0.624
Valvular Disease	1023	7.35	247	7.85	0.328
Weight Loss	32	0.23	8	0.25	0.838
Mental Disorder	987	7.09	215	6.84	0.643
Cancer	209	1.50	49	1.56	0.808
Diabetes	4475	32.16	939	29.86	0.012

Mental disorder: includes depression and psychoses; Cancer: includes lymphoma, metastatic Cancer, andsolid tumor without metastasis; Diabetes includes insulin dependent and independent diabetes.

**Table 10:** NY-SID Incidences of Post-admission Complications before and after Propensity Score Matching.

	Before Propensity Score Matching					After Propensity Score Matching				
	GA(N=13913)		RA(N=3145)			GA(N=3134)		RA(N=3134)		
Variable Label	N	%	N	%	P-value	N	%	N	%	P-value
Mortality	43	0.31	3	0.10	0.035	8	0.26	3	0.10	0.226
Stroke	33	0.24	5	0.16	0.531	4	0.13	5	0.16	1
Paralysis	62	0.45	7	0.22	0.086	12	0.38	7	0.22	0.359
Other Neurologic Disorder	66	0.47	10	0.32	0.299	13	0.41	10	0.32	0.677
Aspiration	73	0.52	6	0.19	0.012	19	0.61	6	0.19	0.014
Respiratory Failure	302	2.17	40	1.27	0.001	49	1.56	40	1.28	0.393
Pulmonary Resuscitation Procedure	178	1.28	17	0.54	<0.001	32	1.02	17	0.54	0.044
Cardiac Arrest	284	2.04	57	1.81	0.438	54	1.72	57	1.82	0.848
Cardiac Resuscitation Procedure	31	0.22	4	0.13	0.384	2	0.06	4	0.13	0.687
Myocardial Infarction	134	0.96	19	0.60	0.059	26	0.83	19	0.61	0.370
Congestive Heart Failure	76	0.55	14	0.45	0.586	10	0.32	13	0.41	0.667

GA: General anesthesia; RA: Regional anesthesia;
